# Native speakers kick buckets, but learners kick doors: A comparison of native and nonnative idiom comprehension

**DOI:** 10.3758/s13421-025-01843-5

**Published:** 2026-01-22

**Authors:** Evelyn Milburn, Mila Vulchanova, Valentin Vulchanov, David Saltzman, James Magnuson

**Affiliations:** 1 Department of Psychology, North Dakota State University, Dept 2546, PO Box 6050, Fargo, ND 58102 USA; 2 Department of Language and Literature, Norwegian University of Science and Technology, Trondheim, Norway; 3 Department of Psychological Sciences, University of Connecticut, Storrs, CT USA; 4 Institute for the Brain and Cognitive Sciences, Storrs, CT USA; 5 Basque Center on Cognition, Brain, and Language, Donostia-San Sebastián, Spain; 6 Ikerbasque: Basque Foundation for Science, Bilbao, Spain

**Keywords:** Idioms, Figurative language, Second language learning, Multiword expressions

## Abstract

Multiword expressions—also called multiword chunks, fixed expressions, lexical bundles, or formulaic sequences—are familiar sequences of words that occur with high frequency in language. Recent focus on multiword expressions, as distinct units of language with distinct processing ramifications, raises the question of how they are used during second language processing: Although processing of multiword expressions appears to grow more native-like as proficiency increases, even highly proficient L2 speakers may not process multiword expressions in entirely native-like ways. We conducted a visual world eye tracking study of idiom comprehension to examine how the balance between compositional and whole-phrase processing changes in second language comprehension. L1 and advanced L2 English speakers listened to sentences consisting of a literal or figurative biasing context clause and a target clause with the final word missing, while simultaneously viewing images representing possible literal or figurative continuations of the clause. Growth curve analysis of eye movement data provided suggestive evidence that L1 and L2 speakers were biased towards different processing strategies during idiom comprehension, with L2 speakers, in particular, taking a more compositional approach towards computing figurative meaning. We also found evidence of qualitative processing differences between literal and figurative expressions, with literal processing being strongly driven by anticipatory mechanisms and figurative processing being driven by within-phrase associations for both groups.

## Introduction

### Multiword expressions in first and second language

Multiword expressions—also called multiword chunks, fixed expressions, lexical bundles, or formulaic sequences—are familiar sequences of words that occur with high frequency in language. Examples include common binominals like *fish and chips*, speech routines like *don’t worry about it*, or figurative expressions like *take the wheel*, to name just a few. Estimates suggest that multiword expressions constitute one-third to one-half of all language (Conklin & Schmitt, 2008) and serve a wide variety of pragmatic functions, such as facilitating social interaction, organizing discourse, or efficiently expressing technical information. Efficiency in recognizing and using multiword expressions is thus an important skill for language users, as multiword expressions are an integral part of everyday speech.

Increasing evidence also shows that multiword expressions serve as distinct units of language and induce processing effects that are separate and distinguishable from the effects of processing their component words. Paralleling results from the processing of single words, comprehenders are sensitive to the frequency of multiword expressions such that higher-frequency phrases receive facilitated processing (Ellis, 2002; Siyanova-Chanturia, Conklin, & Schmitt 2011a, Siyanova-Chanturia, Conklin, & van Heuven, 2011b). Complicating the picture, research on processing of frequent collocations also shows separable effects of single-word and overall phrase frequency (Arnon & Snider, 2010; Öksüz et al., 2020; Tremblay et al., 2011). Taken together, these studies demonstrate that the language comprehension system is sensitive to distributional information at multiple levels (in this case, single words and whole phrases) simultaneously, and illustrate the psychological reality of multiword phrases.

This recent focus on multiword expressions as distinct units of language with distinct processing ramifications raises the question of how they are used during second-language processing. Adult second-language users are able to recognize and use multiword expressions, often with a very high degree of accuracy (González Fernández & Schmitt, 2015; Siyanova & Schmitt, 2008), but their use of these expressions does not appear to directly parallel that of native speakers. Instead, adult second-language users produce fewer multiword expressions than do native speakers, rely on a small number of multiword expressions and tend to ignore the rest, and treat multiword expressions as more syntactically flexible than they actually are (Arnon & Christiansen, 2017; González Fernández & Schmitt, 2015). One possible explanation of these differences is that native and nonnative speakers acquire and process multiword expressions in fundamentally different ways: native speakers are able to bypass compositional processing of multiword expressions and retrieve their meanings directly from the lexicon, whereas nonnative speakers are more biased towards compositional processing (Wray et al., 2016).

However, there is evidence from empirical research which suggests that the balance between compositional and noncompositional (holistic) retrieval changes as nonnative speakers gain more proficiency with the language. Siyanova-Chanturia, Conklin, and van Heuven (2011a) found that L2 users across a range of proficiencies were sensitive to binominal frequency, but only highly proficient users were additionally sensitive to phrase configuration. Consistent with this, Wolter and Yamashita (2018) found that sensitivity to both word frequency and phrase frequency increased with proficiency. They presented native English speakers and higher- and lower-proficiency English learners with adjective–noun collocations and measured reaction times in a speeded acceptability judgment paradigm. Critically, all three participant groups appeared to be sensitive to both word- and phrase-level frequency, as indexed by faster reaction times in response to higher frequency whole phrases and component words. However, whether word or phrase frequency had a stronger effect on reaction times varied depending on participant group. Phrase frequency more strongly influenced reaction times for native speakers and higher-proficiency learners. Word frequency, on the other hand, influenced reaction times for both learner groups, but had less of an effect for native speakers. The difference between groups thus seems to lie in the degree to which each group attends to word- versus phrase-level frequency during meaning retrieval: native speakers are sensitive to word-level frequency but rely primarily on phrase frequency, whereas learners rely on word-level frequency with sensitivity to phrase frequency developing as proficiency increases. Taken together, these studies demonstrate that, despite differences with native speakers, nonnative speakers are sensitive to frequency information at multiple levels simultaneously. Additionally, nonnative speakers are able to use this multilevel frequency information actively during meaning retrieval, depending on level of proficiency.

### Idiom processing

Significant research in the fields of word learning (Grainger et al., 2012; Rau et al., 2014) and literal multiword phrase processing (Conklin & Schmitt, 2008; Ellis et al., 2008; Jiang & Nekrasova, 2007) has investigated how the balance between compositional and holistic processing changes as a learner becomes more proficient. One particularly useful way of examining this balance is by examining how L2 learners comprehend and process idioms. Idioms are conventionalized multiword expressions in which the literal meanings of the expression’s component words do not (always) directly contribute to the meaning of the expression, resulting in a figurative or nonliteral interpretation (Gibbs, 1980; Glucksberg, 1991). The difference between these literal and figurative meanings can be stark: for example, the idiom *turn the tables* figuratively means “to move from a weak position to a stronger one,” but can also be interpreted literally as “to rotate some tables.” This presents an interesting conflict between compositional and holistic meanings that the language comprehension system must solve, if an accurate interpretation of the phrase is to be reached.

Most empirical research on idiom comprehension and processing can be roughly divided into two streams—one broadly concerned with the activation of idiom form and the other broadly concerned with the construction of idiom meaning given a particular context or idiom-internal characteristic (for a review, see Senaldi & Titone, 2022). For example, upon encountering the multiword phrase *turn the tables*, a researcher interested in the activation of idiom form might ask if and how retrieval of this phrase differs from the matched literal phrase *set the tables*. In contrast, a researcher interested in the construction of idiom meaning might ask how the context in which *turn the tables* is embedded influences whether or not a comprehender is likely to interpret the phrase literally or figuratively. These two research streams are difficult to disentangle from each other, as the processes of idiom form activation and idiom meaning construction are intertwined, and researchers (rightfully) have not strictly differentiated between them in previous work. In this section, we review fundamental theories of idiom form activation as they relate to our research question of compositional versus holistic processing in native and nonnative speakers.

Early models of idiom recognition and comprehension posited an entirely holistic comprehension process: idiom meanings are directly retrieved from memory with no compositional processing of their component words (Bobrow & Bell, 1973; Gibbs, 1980). These are models such as Swinney and Cutler’s (1979) lexical representation hypothesis or Gibbs’ (1980) direct access model. Under these early models, idiom comprehension proceeds as the result of a distinct figurative comprehension process, distinguishable from the process of comprehending literal language. Such models assume that idioms are represented as groups of words or “long words,” which are subsequently directly accessed and retrieved from memory. These models capture an essential component of idiom comprehension and processing in their focus on the phrasal nature of idioms.

However, later evidence showed that individual component words do have measurable effects on idiom comprehension and processing. For example, Gibbs and colleagues (1989) demonstrated that many idioms are both lexically and syntactically flexible, meaning that their lexical makeup and syntactic form can be changed without altering the figurative meaning. For example, the idiom *let off some steam* can be lexically altered to *let off some heat* or syntactically transformed to *steam was let off* without meaningfully altering its figurative interpretation. This pattern would not be predicted if idioms were exclusively retrieved from memory as a fixed form with no processing of their component words. Likewise, Hamblin and Gibbs (1999) showed that the lexico-semantic properties of verbs in idioms constrained the contexts in which idioms were considered acceptable: *kick the bucket* describes a fast death rather than a slow one because of the punctual nature of the verb *kick*.

These and other lines of research have led to the important observation that idioms do not form a single homogenous class, with similar processing effects elicited across all extant idioms. Rather, much research and theoretical discussion has focused on classifying idioms into different types (e.g., Nunberg et al., 1994), and on examining processing ramifications between types. For example, significant research has also focused on processing of decomposable idioms, or idioms in which an idiom’s component words are related to or correspond in some way to the idiom’s overall figurative meaning. Idioms like *break the ice* or *sing the blues* are decomposable: *the ice* is an awkward social situation, and *breaking* is the act of making that situation relaxed and comfortable. In contrast, idioms like *kick the bucket* or *chew the fat* are generally characterized as nondecomposable. Decomposable idioms are typically processed faster than nondecomposable idioms, which would again not be predicted if idiom processing was entirely holistic and relied on lexical storage. Other research has investigated the similar construct of transparency, or the degree to which a comprehender can make a sensible connection between an expression’s literal and figurative meaning (Keysar & Bly, 1995). Recent research, however, has indicated that constructs like decomposability and transparency are inherently unstable, with ratings varying significantly between participants (Carrol & Littlemore, 2020; Nordmann, et al., 2014). Moreover, it is not always clear what is being measured when participants are rating decomposability and transparency (Griffen & Noveck, 2025; Skoufaki, 2008), with evidence from research that these constructs may be colinear. The relationship between single word and overall phrase meaning in idiom processing is thus a complex one. This complexity produces further difficulty for the idiom researcher, who must not assume that all idioms elicit the same processing mechanisms while remaining alert to potential similarities across idiom types.

Adding to this complexity, idioms do not seem to be processed entirely compositionally either: idioms are typically processed faster than matched compositional phrases (Carrol & Conklin, 2020; Conklin & Schmitt, 2008; Libben & Titone, 2008; Tabossi et al., 2009), suggesting that their formulaic nature confers processing advantages associated with direct retrieval of the whole idiom from memory (cf. Gibbs, 1980; Swinney & Cutler, 1979). This tension between direct retrieval and compositional processing is reflected in current models of idiom recognition and processing. These models typically include mechanisms to support either simultaneous or sequential compositional processing and direct retrieval. For example, in Titone and Connine’s (1999) *hybrid model*, direct retrieval and compositional processing proceed simultaneously. Cacciari and colleagues (Cacciari & Glucksberg, 1991; Cacciari, & Tabossi, 1988) posit a slightly different view in their *configuration hypothesis*: Idiom processing proceeds compositionally and literally until the statistical co-occurrence frequencies of the words within the idiom signal that an idiom has been encountered (this recognition point is referred to as the “idiom key”), at which point compositional processing is abandoned and the idiom meaning is directly retrieved.

A related model assumes both direct access and compositional processing, albeit on different time-scales. Titone and colleagues (2019) asked participants to read idioms and matched literal phrases (*had a lark* vs. *saw a lark*), followed by a literal or figurative disambiguating region. They found early advantages for idiomatic forms with no interactions with either familiarity or decomposability, suggesting that early stages of idiom processing involve direct retrieval of whole idiom forms (consistent with both the *hybrid model* and the *configuration hypothesis*). They therefore suggest that compositional analysis and idiom decomposability are mostly relevant in later stages, when a literal or figurative idiom meaning is being selected and integrated with context. Taken together, there is evidence consistent with both direct retrieval and compositional analysis during idiom comprehension, with phrase frequency and idiom decomposability being major factors governing whether a particular idiom will be processed in a way seemingly consistent with lexical retrieval versus compositional analysis, at least during comprehension by native speakers.

Evidence from L2 idiom processing and comprehension, however, supports the view that this process may be biased towards compositional analysis of individual words rather than holistic retrieval of whole phrases for later learners. Although nonnative speakers are certainly able to process figurative meanings of idioms in their L2 (Beck & Weber, 2016; van Ginkel & Dijkstra, 2019), there appears to be a gulf between L2 speakers’ knowledge about what an idiom means and their ability to quickly and smoothly retrieve idiom forms from the lexicon. For example, Siyanova-Chanturia, Conklin, and Schmitt (2011b) asked native and nonnative speakers to read idioms embedded into contexts biasing their literal or figurative meanings (e.g., *at the end of the day* meaning “in the evening” vs. “eventually”) and compared them to matched compositional phrases (*at the end of the war*). In contrast to native speakers, non-native speakers showed no advantage for idioms over compositional phrases and read idioms more slowly when they appeared in figuratively biased contexts. Siyanova-Chanturia and colleagues interpreted this pattern of results as reflecting a bias towards compositional, literal, processing, perhaps as a result of weaker connections between the whole-phrase form of an idiom and its figurative meaning.

Similarly, Carrol and colleagues (2016) found that L1 and advanced L2 speakers performed comparably well on late eye-tracking measures when reading idioms, suggesting that figurative meanings were available to both populations with a high degree of facility. However, L2 speakers showed deficits in early measures of processing on idioms’ final words (e.g., likelihood of skipping final words), suggesting that lexical combinations were not as well entrenched in the lexicons of L2 speakers, even though their meanings were available. In consideration of these results, Carrol and colleagues point out that, although truly “nativelike” idiom processing is a high bar to clear, nonnative speakers are certainly able to understand and process idioms, often with a correspondingly high degree of ease and rapidity. This analysis is consistent with findings that non-native speakers can process both literal and figurative meanings of idioms, but that their intuitions about idiom properties (i.e., decomposability or transparency) are less well-developed than those of native speakers (van Ginkel & Dijkstra, 2019), as well as findings that the balance between compositional and holistic idiom processing changes based on the comprehender’s language dominance (Cieślicka & Heredia, 2017). Further evidence supporting a difference in intuitions between L1 and L2 speakers comes from the study by Singstad (2014). The design involved an intervention study targeting idiom identification and practice with deriving idiomatic meanings in advanced high-school Norwegian L2 learners of English. The intervention produced a significant difference between the control group and the intervention group on both idiom identification and an idiom meaning test. In addition, the intervention component also showed that L2 learners have a problem initially identifying phrases as idiomatic and, consequently, acquiring their figurative meaning.

Supporting this perspective, Senaldi and Titone (2022) recently demonstrated L2 speakers’ bias towards compositional processing in an eye-tracking study of idiom reading. In their experiment, French–English bilinguals read English sentences containing idioms used either literally or figuratively (*Dolan spilled the beans when he mentioned the surprise party to his friend* vs. *Dolan spilled the beans when he tried to pour too many into the soup pot*), or matched literal control phrases. They found slower reading times on the idioms and on the idiomatic disambiguating regions, specifically for idioms with no L1 equivalent and which were non-decomposable. This pattern of results suggested that early processing of L2 idioms is primarily compositional, with whole-phrase retrieval and decomposability effects kicking in (where possible) along different time scales to aid processing. In a follow-up experiment, the authors disrupted direct retrieval of idiomatic forms by introducing a language-switching manipulation, wherein the last word of an English idiom was switched to French (e.g., *Dolan spilled the* fèves). They found no specific slowdown in reading measures for switched idioms compared to switched matched control phrases (e.g., *Dolan cooked the* fèves), indicating that readers appear to be parsing both conditions compositionally. This is suggestive evidence that second language learners tend to rely on compositional processing over whole-phrase retrieval during idiom comprehension (this perspective is also broadly compatible with the proposal that L2 speakers prioritize literal meanings due to their greater salience; Giora, 2002).

### Context in idiom processing

As discussed in the previous section, one major stream of idiom research has investigated the construction of idiom meaning, or how contextual or idiom-internal characteristics might push a comprehender to interpret a phrase literally or figuratively. In this section, we discuss research showing that contextual information can help a comprehender select the appropriate meaning of a figurative phrase, and that context is especially important in scaffolding figurative interpretation for nonnative speakers (for an overview, see Vulchanova et al., 2019).

A long history of research shows that figurative idiom meanings are processed more easily when embedded in supportive contexts (Beck & Weber, 2016; Gibbs, 1980; Holsinger & Kaiser, 2013; Ortony et al., 1978; Qualls et al., 2003; Titone & Connine, 1999). Context can likewise push idiom interpretation towards the literal (i.e., towards a “rotate the tables” interpretation of *turn the tables*) with little or no processing slowdown. In an eye-tracking study of reading, Milburn and Warren (2019) preceded idioms with either literally or figuratively biased contexts, finding no difference in eye movement measures regardless of context for L1 speakers. One interpretation of these results is that participants used context to select the biased meaning of the idiom with no evidence of interference from the unbiased meaning (for a similar study also finding no evidence of interference from unbiased meanings, see Siyanova-Chanturia, Conklin, and Schmitt, 2011a). This pattern of results is strong evidence that context is an important scaffold for choosing a particular idiom meaning among alternatives for native speakers.

Context is especially important for supporting figurative idiom interpretation in L2 learners. In general, supportive context facilitates figurative idiom interpretation. In an offline study of idiom comprehension, Liontas (2003) found that L2 learners of Spanish were better able to state the meanings of Spanish verb-phrase idioms (*está colgando de un hilo*/“he’s hanging on by a thread”) when they appeared in supportive contexts than when they appeared in isolation. Online comprehension measures have likewise found processing facilitation for supportive contexts. In their study of L2 learners’ eye movements in reading, Cieślicka and colleagues (2014) found that total reading times on idioms were shorter when idioms were preceded by a supportive (vs. neutral) context, and additionally that post-idiom regions were fixated and regressed from less frequently. Beck and Weber (2020) likewise found that supportive contexts boosted figurative idiom interpretation in self-paced reading, although a similar boost was not observed for idioms used literally (e.g., *break the ice* when used to literally mean shatter ice). These results show that context supports figurative meaning access in L2 learners in similar ways to native speakers, with figurative interpretations accessed more easily and integrated more quickly when scaffolded by supportive context.

## Experiment

The present study asks two questions at the intersection of idiom form retrieval and idiom meaning computation. First, we ask whether figurative expressions are activated and retrieved qualitatively differently than literal expressions with similar collocational frequency. Second, we ask whether L1 and L2 speakers rely on different strategies (compositional processing vs. holistic retrieval) when accessing idiom forms (Senaldi & Titone, 2022). Using the visual world eye-tracking paradigm (Tanenhaus et al., 1995), L1 and proficient L2 English speakers listened to sentences consisting of a biasing context clause and a target clause with the last word missing, while simultaneously viewing images representing possible literal or figurative continuations of the clause. Whether literal or figurative interpretation was appropriate depended on the context clause. We also included a neutral context condition. Given extant evidence, we expected that L1 and proficient L2 speakers would be biased towards different processing strategies during idiom recognition. We expected that L1 speakers would be biased towards whole-phrase idiom retrieval, especially at early stages of processing, and show less reliance on context, indexed by increased proportion of fixations towards figurative completions in literally biased contexts. In contrast, we expected that proficient L2 speakers would be biased towards literal, compositional processing and would be more reliant on context, indexed by fixation proportions to figurative completions only in figurative contexts.

## Methods

### Participants

#### L1 speakers

Forty-seven undergraduate native English speakers participated for course credit at the University of Connecticut. Participants had normal or corrected-to-normal vision and did not participate in the stimulus norming described below. All procedures were approved by the University of Connecticut’s IRB. Participants received partial course credit for participating, but participation was voluntary in that students could choose options other than participating in experiments.

#### L2 Speakers

Forty-eight undergraduate native Norwegian speakers participated (note that the same data from these participants reported here were previously reported in Milburn et al., 2021). Participants began learning English at an average age of 6.06 years (self-report), were native speakers of Norwegian who had grown up in Norway, had normal or corrected-to-normal vision, and did not participate in the stimulus norming described below. Ethics approval was received from the Norsk Senter for Forskningsdata (Norwegian Center for Research Data). Participants received a gift card for 100 kroner (~$10) for participating.

### Materials

**Fig. 1:**
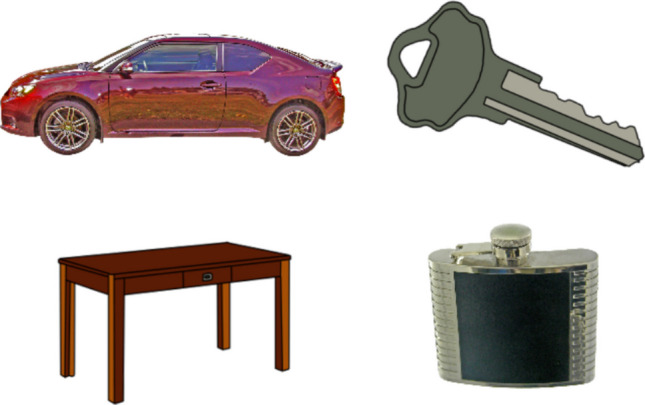
Prototypical image array for example idiom *turn the tables,* showing the figurative target (table), literal target (car), high-collocation lure (key), and low-collocation lure (flask). This visual display was accompanied by the auditory stimuli 1a–c, shown in the text

Visual stimuli consisted of 21 arrays of four images arranged in a grid. For example, one prototypical array (see Fig. 1), for the idiom *turn the tables*, consisted of a table, a car, a key, and a flask. Images were selected from either the MultiPic databank (Duñabeitia et al., 2018) or the Bank of Standardized Stimuli (Brodeur et al., 2014).

Each array was accompanied by one of three possible sentence fragments consisting of a biasing context clause (not included in the unbiased condition described below), an agent, and an idiom region with the final object argument missing. Participants were instructed to click on the image that best completed the sentence (see Kessler et al., 2020, for a similar design). Final clauses could be completed to form either literal or figurative phrases; for example, “turn the” can be completed literally as “turn the car” or figuratively as “turn the tables” (where the noun phrases “the tables” and “the car” have the same frequency of occurrence after the verb “turn”). We will refer to this final clause as the “idiom region,” although idiomatic interpretation was not always required. The words before the idiom region, referred to as the pre-idiom region, were the same across conditions, although the length of this region varied between items. Context clauses biased either a figurative completion or a literal completion, or were unbiased (1a–c; pre-idiom region underlined; idiom region bold). The unbiased condition usually consisted of the pre-idiom region and the idiom region only.

1a. (Figuratively biased) To get even with his enemies, Chia-Ming **turned the** ____

1b. (Literally biased) To get to his destination, Chia-Ming **turned the** ____

1c. (Unbiased) Chia-Ming **turned the** ____

All images in the array were possible continuations of the sentence but varied in how compatible they were with the biasing context. The figuratively biased context (1a) was most compatible with the *table* in Fig. 1, referred to as the *figurative target*: the idiom “turn the tables” means to move from a weak position to a strong one. The literally biased context (1b) was most compatible with the *car* in Fig. 1, referred to as the *literal target*. The unbiased context (1c) was most compatible with the *key* in Fig. 1, referred to as the *high-collocation lure*, although all images were possible continuations in this condition. Finally, *low-collocation lures* (like *flask* in our example) were most compatible with the unbiased context (though their mean collocation was lower than the means for the literal and figurative targets; see Table 1).

**Table 1:** Mean frequency per million and standard deviation (*SD*) of the four possible sentence continuations in the Corpus of Contemporary American English

Image	Mean (COCA freq. per mil)	*SD*
Figurative target (table)	.05	.05
Literal target (car)	.04	.05
High-collocation lure (key)	.23	.28
Low-collocation lure (flask)	.003	.01

Note that, although targets were *most* compatible with their appropriate biasing context, they were not incompatible with other contexts (see Kyriacou & Köder, 2024, for a discussion). Rather, contexts were biased such that the appropriate target was strongly favored as a completion and other objects were disfavored, as established by norming. Fixation proportions to the appropriate target in a biasing context condition therefore reflect gaze driven by true contextual likelihood, rather than the elimination of other possibilities. Note also that the purpose of the neutral context condition was to check for possible saliency differences in our visual stimuli that might drive eye movements over and above the effects of biasing auditory contexts. If there existed picture categories with intrinsically more saliency, we would expect them to garner more fixations in the neutral context condition as well as in either literal or figurative context conditions. If no differences between looks to different images emerge in the neutral context condition, we can be confident that gaze patterns in the literal and figurative bias conditions are driven by processing of the auditory linguistic stimuli. See the Appendix for a list of all linguistic stimuli.

This paradigm is adapted from one used by Mack et al. (2013; see also Hayes et al., 2016). Mack and colleagues (2013) used a modified version of the visual world paradigm in their study of verb-argument prediction in older adults and people with aphasia. They presented participants with sentence fragments with the final word missing and asked them to indicate which of an array of images on a computer screen best completed that fragment. Critically, because the direct object was not mentioned in any of the sentences, bottom-up information did not confirm the direct object’s identity. This enabled Mack and colleagues to observe any potentially delayed or reduced prediction effects. This paradigm is likewise well-suited to examination of idiom processing in second language learners, who may also exhibit slowed or reduced figurative language processing.

For consistency, idioms largely consisted of a verb, determiner, and noun (e.g., *hold the key*, *wear the pants*), although some varied in structure (e.g., *fall off the map, run out of juice*). However, the final word in all idioms was a noun. We additionally selected English idioms that had no Norwegian equivalent, following findings that idioms that occur in both the L1 and L2 are processed more easily (for a review, see Carrol et al., 2018).

To control for the role of frequency and probability of occurrence in the same configuration (see Vulchanova et al., 2019, for recommendations and a theoretical discussion), the figurative and literal targets were matched in collocation frequency using the Corpus of Contemporary American English (COCA; Davies, 2009). Using COCA’s Collocates function, we searched for noun collocates of the idiom with the final word missing (i.e., noun collocates of “turned the”), spanning two words to the right. For literal targets, we selected noun collocates that were concrete, imageable, and were similarly frequent to the figurative target. For example, “turned the *tables*” and “turned the *car*” have COCA frequencies per million of 0.19 and 0.17, respectively. We also selected high and low collocation frequency lure items. “Turned the *key*” has a COCA frequency per million of 0.4, but “turned the *flask*” has a COCA frequency per million of 0.01. A full list of stimuli is shown in the Appendix. Average frequencies per million for each possible sentence continuation are shown in Table 1.

Audio stimuli were recorded by a female native speaker of English with a sampling rate of 44.1 kHz and a bit rate of 16 bit/s. Critical timepoints were measured by the same native speaker of English. Idiom region audio durations did not differ across conditions, *F*(2,40) =.228,* p* =.79. However, the audio duration of the pre-idiom region differed across conditions, *F*(2,40) = 9.51, *p* <.05. The pre-idiom region in the unbiased condition was significantly longer than pre-idiom regions in both the figuratively biased, *t*(20) = −3.26, *p* <.05, and literally biased, *t*(20) = −5.12, *p* <.05, conditions.

There were 45 trials in the experiment (21 experimental trials and 24 filler trials). Experimental trials were counterbalanced such that participants saw only one condition for each item. We based stimuli selection on stringent criteria consistent with a theoretical proposal developed in Vulchanova et al. (2019), whereby idiom constituents compete (for processing) with the most common nonidiomatic fillers in the same syntactic position. After identifying a possible set of items of the appropriate verb+determiner+concrete noun type, idioms were located in COCA and appropriate high, low, and frequency-matched concrete nouns were identified. Following this narrowing of the potential item set, target and distractor images were located in our selected image databases (Brodeur et al., 2014; Duñabeitia et al, 2018). The item set was narrowed further by the requirements that a) idioms be familiar to both American and Norwegian participants, and b) idioms not appear verbatim in the Norwegian language (e.g., the idiom *take the cake* appears in Norwegian, but means the exact opposite of the English idiom, making it unsuitable for our purposes). Given these constraints, in our view the number of stimuli approaches the upper limit of what is possible for the number of items while respecting these selection/norming constraints.

### Norming

Stimuli were normed to ensure that idioms were familiar to participants, that sentences were similarly natural across conditions, that picture stimuli were identifiable, and that contexts appropriately constrained selection of the final word. All norms were completed by both L1 and L2 speakers (none of whom participated in the subsequent eye-tracking experiment). Idioms were counterbalanced such that participants did not rate the same idioms on both the naturalness and picture norms. All participants rated all idioms on familiarity. All ratings were conducted in English. 20 native speakers of Norwegian living in Norway and 20 native speakers of English living in the United States participated in the norming procedure.

#### Familiarity rating

Participants rated how familiar they were with 31 idioms using a 1–7 Likert scale (1 = *very familiar*; 7 = *very unfamiliar*). Although both groups rated the selected idioms as very familiar, they were more familiar for the native (*M* = 1.86, *SD* =.57) than for the nonnative (*M* = 2.54, *SD* =.83) speakers, *t*(20) = 3.45, *p* <.05.

#### Naturalness rating

Participants were shown each sentence stimulus truncated before the idiom and rated its naturalness on a 1–7 Likert scale (1 = *very natural*; 7 = *very unnatural*). Participants rated only the two biasing context conditions, and stimuli were counterbalanced such that each participant only saw one condition per item. There was no difference in naturalness between conditions for both native (paired-sample *t* test), *t*(20) =.27, *p* =.79, and nonnative, *t*(20) =.89, *p* =.38, speakers.

#### Picture rating

This norm ensured both that stimuli were identifiable and that contexts appropriately constrained selection of the final word. Participants saw each grid of images paired with either the literally biased or figuratively biased sentence fragment, counterbalanced such that participants only saw one version of each item. Participants ranked the images in the grid in order of how well they completed the accompanying sentence. For figuratively biased contexts, participants ranked the figurative target first in 91% (native speakers) and 80% (nonnative speakers) of trials. This indicates that participants had a strong preference that the figurative target was the best completion for the figuratively biased context. For literally biased contexts, participants ranked the literal target first in 86% (native speakers) and 78% (nonnative speakers) of trials, again indicating a strong preference for the literal target as the best completion in the literally biased context.

### Procedure

#### Individual difference measures

L2 English speakers completed two assessments of English L2 skills. The first was LexTALE (Lemhöfer & Broersma, 2012), a test of vocabulary knowledge administered as an unspeeded lexical decision task. Scores on the LexTALE are expressed in percentage correct. LexTALE scores have been shown to be a good predictor of general English vocabulary knowledge and have been used in many studies as indicators of general L2 proficiency. Although single-word and idiom proficiency likely do not follow the same acquisition trajectory, and this goes double for second language acquisition, Carrol (2023) points out an interesting relationship between education, vocabulary size, and idiom knowledge: Although education may predict idiom knowledge in models on its own, it ceases to be predictive once vocabulary size is added to the model. Carrol (2023) interprets this as indicating that vocabulary size is predictive of idiom knowledge independently from education level. Moreover, Carrol (2023) additionally argue that vocabulary size is a good predictor of familiarity (compared with other idiom characteristics, like transparency). We made efforts to ensure that our stimuli would be familiar to both American and Norwegian participants. This is because if idioms were not familiar, we could not be sure that we were measuring figurative processing. Although the selected idioms were more familiar to our American participants (*M* = 1.86, 1–7 scale, with 1 being *very familiar*) than to our Norwegian participants (*M* = 2.54), both groups found them very familiar. We take this and the above point as supporting our use of the LexTALE as an exploratory individual difference measure.

Participants scored an average of 82.98 on the LexTALE (range: 62.50–98.75). Participants also completed a 15-question multiple-choice grammar test conducted through Exam English (examenglish.com). Upon completion of this assessment, participants were classified according to the six levels of the Common European Framework of Reference for Languages (A1–C2). Participants scored overall very high on this measure (range: B2–C2; Mode: C2). See the Discussion section of the present paper and Milburn et al. (2021) for more discussion of these measures.

#### Eye tracking

Participants’ eyes were tracked using a desktop-mounted EyeLink 1000 tracker (SR Research Ltd., Toronto, Ontario, Canada) with a sampling rate of 2 ms and a spatial resolution of less than a 30-min arc. The experiment was programmed and presented using the Experiment Builder software (SR Research Ltd., Toronto, Ontario, Canada). Participants viewed stimuli binocularly on a monitor approximately 68 cm from their eyes. Head movements were minimized using forehead and chin rests. After explaining the instructions to the participants, we calibrated the eye tracker using a 9-point fixation grid; this ensures that the eyes are tracked both precisely and accurately across the entire display screen. In each trial, participants first clicked on a centrally located fixation cross, thereby repositioning eye gaze and mouse location at the center of the screen. The image array was displayed, followed 1,500 ms later by the audio stimulus. Participants clicked on the image that best completed the sentence. After each trial, a single-point, centrally located drift check was performed to assess whether recalibration was necessary. Full recalibrations were performed as necessary. The positions of the images within the array were randomized, as was the order in which stimuli were presented. Audio stimuli were presented to participants via headphones (native speakers) or by two loudspeakers positioned at either side of the viewing monitor (nonnative speakers). The experiment lasted approximately 20 min.

#### Open science practices

All (deidentified) data and analysis scripts are available online (https://osf.io/7cyus/overview). This study was not preregistered.

## Results

### Mouse-click accuracy

**Fig. 2:**
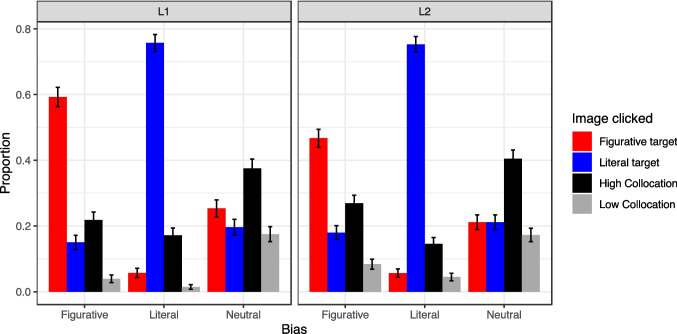
Average mouse-click selection of the four images in each condition. (Color figure online)

Proportions of mouse selections in different bias contexts are shown in Fig. 2. The key questions are whether processing differs for figurative versus literal bias contexts, and whether the L1 and L2 samples differ. To address this, we constructed a linear mixed-effects model examining mouse presses of the expected targets for the figurative bias context (proportion of clicks to the figurative target) and the literal bias context (proportion of clicks to the literal target). We used the *lme4* package (Bates et al., 2015) for R (R Core Team, 2023) and specified the model as in Equation 1 (where pExpected is proportion of clicks on the expected target).

1 $$\text{pExpected }\sim \text{ Bias }\times \text{ Population}+\left(1 |\text{ Subject}\right)$$

Results are summarized in Table 2. We used treatment coding, so the intercept value represents the mean proportion of expected target clicks for sample L1 in the figurative bias context. The effect of bias indicates that the proportion was significantly (17%) higher in the literal bias context compared with the figurative bias context. The effect of Sample shows that the L2 sample had significantly (12%) lower target clicks than L1. Finally, the significant interaction of bias and sample indicates that the combined impact of being in the literal bias condition and L2 sample was significantly higher than expected from the individual effects of bias and sample. Or more simply, we can see in Fig. 2 that L2 participants had lower figurative click proportions in the figurative bias condition than L1 participants, but virtually identical literal click proportions as L1 participants in the literal bias context.

**Table 2:** Comparison of clicks to expected targets in the figurative and literal bias contexts for samples L1 and L2

*Predictor*	*Estimates*	*CI*	*t*	*p*
(Intercept)	0.57	0.51, 0.63	**19.29**	**<.001**
Bias [Literal]	0.17	0.09, 0.25	**4.11**	**<.001**
Sample [L2]	−0.12	−0.20, −0.04	**−2.98**	**.003**
Bias [Literal] × Sample [L2]	0.12	0.01, 0.23	**2.15**	**.033**

**Fig. 3:**
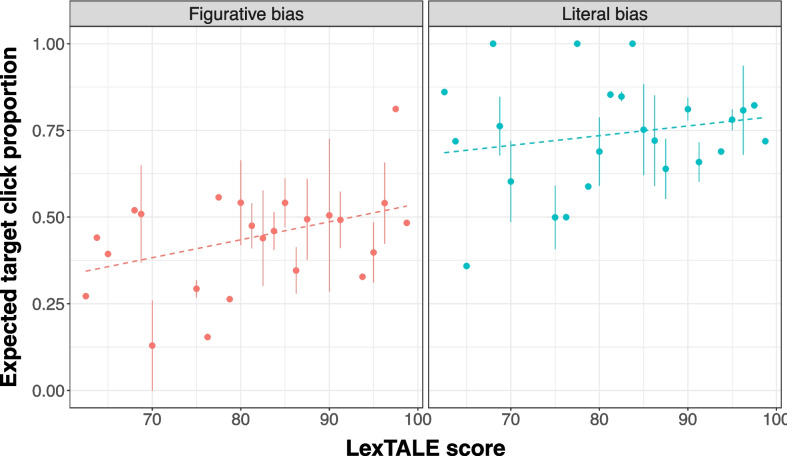
Points indicating proportion of clicks on expected targets in the figurative and literal bias contexts for L2 listeners are compared to the LexTALE proxy for proficiency. Lines indicate model fits

We also examined individual differences by adding LexTALE score as a predictor in a model only including the L2 sample. The mixed-effects model results are summarized in Table 3. Within the L2 group, there is a nonsignificant trend for greater proportion of clicks in the literal bias context, and a nonsignificant trend for LexTALE score to predict proportion of clicks on expected targets. These results are visualized in Fig. 3. The trends are interesting, but would have to be followed up with a subsequent study with a larger sample and possibly a larger range of L2 proficiency to be truly informative.

**Table 3:** Individual differences in mouse clicks

*Predictor*	*Estimate*	*CI*	*t*	*p*
(Intercept)	0.02	−0.44, 0.48	0.09	0.929
Bias [Literal]	0.49	−0.08, 1.06	1.70	0.092
LexTALE	0.01	−0.00, 0.01	1.88	0.064
Bias [Literal] × LexTALE	−0.00	−0.01, 0.00	−0.69	0.493

#### Fixation proportion results

**Fig. 4:**
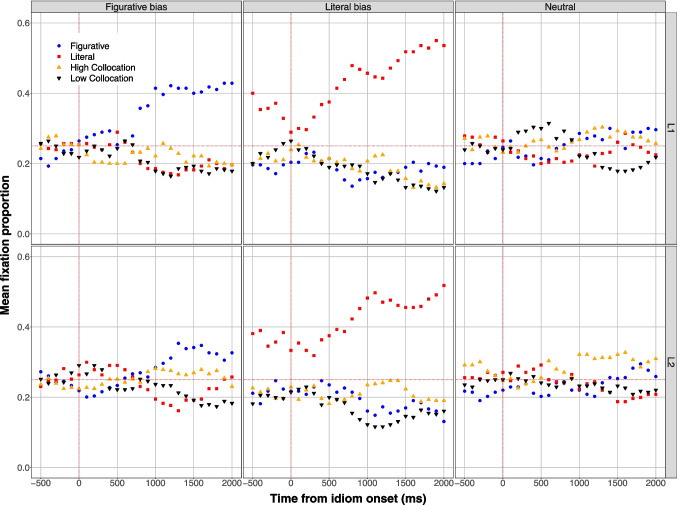
Fixation proportions over time to item types in bias contexts (columns) by sample (rows). (Color figure online)

Fixation proportions over time to the four item types in the three biasing conditions for the two language samples are shown in Fig. 4. Here, we present the full time range for which we have data (from 2,000 ms prior to idiom onset to 3,000 ms after). Salient differences are apparent between bias contexts. The literal bias context stands out: both L1 and L2 participants showed very strong target anticipation in this condition, as well as the highest peak fixation proportions of any context for this condition’s expected target (the literal target). In contrast, in the figurative bias context, fixations to the expected (figurative) target did not separate from fixations to other items until substantially after the idiom onset, and had lower peaks than literal targets in the literal bias context. Finally, the neutral bias context also stands out as strikingly different from the other two, in that no item type showed a strong peak. That said, there is an apparent trend for L2 participants to fixate the expected (high collocation) target more in the late time course (though as can be seen in Fig. 2, they were not robustly more likely to click on that item than L1 listeners). Thus, the neutral bias context provides a confirmation that neither the literal nor figurative target items were unusually salient. That is, if participants were more likely to fixate those items in the neutral bias context than the high collocation lure in particular, that would raise questions about preferences for those items in the other contexts. Since we see no evidence for such problematic preferences, we will not discuss the neutral bias condition further.

To assess our two primary research questions (whether figurative [idiomatic] and literal expressions are processed qualitatively differently, and whether L2 listeners differ from L1 listeners when processing such expressions), we compared expected target fixation proportions in the figurative and literal bias contexts using a growth curve analysis (GCA) approach (Magnuson et al., 2007; Mirman, 2014; Mirman et al., 2008). The GCA approach is best suited towards comparing over-time changes in fixation proportions for relatively few item types and relatively few conditions. It is possible to conduct more complex analyses, but with increased complexity, models become more difficult to interpret. Focusing just on expected targets (i.e., the figurative and literal targets, respectively, in the figurative bias and literal bias contexts) provides a compact, interpretable, and sufficient approach to address our core questions.

The anticipation results observed in the literal bias condition were unexpectedly large. They also suggest, at least for the L1 group, two distinct phases: one of anticipation driven by the context sentence, and one driven by the actual target expression (aligned at “idiom onset”). While we could model the entire time series, there is no advantage to doing so. On the GCA approach, the anticipation window will not be demarcated from the post-idiom onset window, and so, for example, differences between L1 and L2 participants specific to those phases will likely manifest as intercept and slope differences over the entire (−2,000 to +3,000) window. Hence, we will conduct separate analyses on the post-idiom offset window, and then the anticipation window.

**Post-idiom onset fixation proportions.** This analysis window began at idiom onset and ended 3,000 ms post-idiom onset. For this window, we began with a maximal model with four over-time (*ot*) parameters (i.e., orthogonal power polynomials corresponding to slope, quadratic, cubic, and quartic terms). Equation 2 provides the details for the model specification we used with the R package *lme4* (Bates et al., 2015) to assess mean fixation proportions to expected targets as a function of the four *ot* parameters as a set, main effects of *bias* (figurative, literal) and *sample* (L1, L2), and two- and three-way interactions between each *ot* term, *bias*, *sample*, along with maximal random effects structure (note that the “(2)” to the right is the equation number, not part of the equation).

2 $$\begin{array}{l}\text{meanFix }\sim (\mathrm{ot}1 +\text{ ot}2 +\text{ ot}3 +\text{ ot}4) \times \text{ Bias }\times \text{ Sample}+\\ (\mathrm{ot}1 +\text{ ot}2 +\text{ ot}3 +\text{ ot}4 |\text{ Subject}) +\\ (\mathrm{ot}1 +\text{ ot}2 +\text{ ot}3 +\text{ ot}4 |\text{ Subject}:\mathrm{Bias})\end{array}$$

**Fig. 5:**
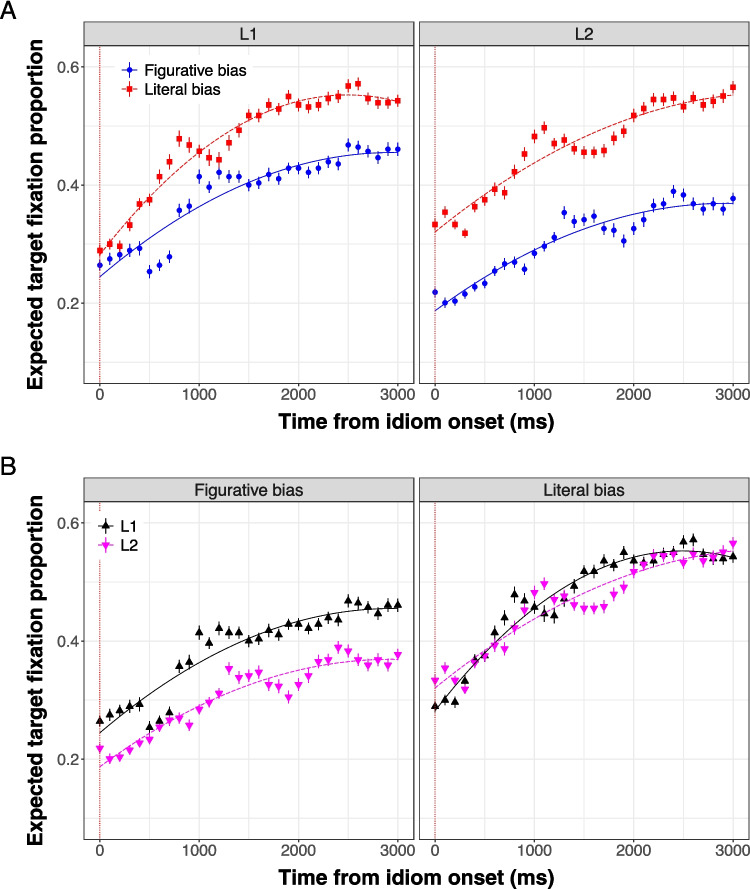
Expected target fixation proportions (points) compared with GCA fits (lines). Panel **A** plots points and lines for figurative targets given figurative bias (blue) and for literal targets given literal bias (red) for L1 and L2. Panel **B** shows the same data, but comparing figurative targets given figurative bias (left) for L1 and L2, and then literal targets given literal bias (right) for L1 and L2. (Color figure online)

*Bias* (figurative, literal) and *sample* (L1, L2) were both sum-coded for contrasts (rather that the default of treatment coded) to facilitate interpretation of results. This maximal model did not converge; however, the model results indicated that there were no main effects or interactions associated with either *ot3* or *ot4*. Therefore, rather than attempting to simplify the random effects structure, we dropped the *ot4* term and attempted a maximal model with just three *ot* terms (the same as the equation above with each instance of *ot4* removed). Using *allFit*, we were able to obtain *lmer* settings for this model that allowed it to converge. As expected, it was still the case that the main effect of *ot3* was not significant and that there were no significant interactions involving *ot3*. Thus, we further simplified the model, removing all instances of *ot3* from the equation above. The *ot2* model results are summarized in Table 4 (note also that the patterns of significance for all remaining predictors were the same in the models including *ot3* and *ot4* terms).^1^ Figure 5 compares GCA fits with observed mean fixation points in the post-idiom onset window.

**Table 4:** Post-idiom onset GCA results for expected target fixation proportions in figurative and literal bias conditions for L1 and L2 samples

*Predictor*	*Estimate*	*CI*	*t*	*p*
(Intercept)	0.39	0.35, 0.42	21.476	**<.001**
*ot1*	0.48	0.31, 0.65	5.461	**<.001**
*ot2*	−0.13	−0.24, −0.02	−2.315	**.021**
Bias [Literal]	0.09	0.04, 0.14	3.749	**<.001**
Sample [L2]	−0.08	−0.12, −0.03	−3.129	**.002**
*ot1* × Bias [Literal]	0.11	−0.12, 0.35	0.931	.352
*ot2* × Bias [Literal]	−0.10	−0.25, 0.04	−1.440	.150
*ot1* × Sample [L2]	−0.06	−0.30, 0.17	−0.535	.592
*ot2* × Sample [L2]	0.02	−0.13, 0.17	0.226	.822
Bias [Literal] × Sample [L2]	0.07	0.00, 0.13	2.024	**.043**
(ot1 × Bias [Literal]) × Sample [L2]	0.00	−0.32, 0.32	0.006	.995
*(ot2* × Bias [Literal]) × Sample [L2]	0.11	−0.08, 0.30	1.134	.257

In Table 4, the significant intercept and *ot* terms characterize how the mean pattern differs from a flat line with intercept 0. In GCA with power polynomials, the intercept term indicates the mean centered intercept (thus, the mean difference from 0). *ot1* indicates overall linear slope, while *ot2* indicates (quadratic) bowing. The main effects of bias and sample are also significant, indicating more fixations to the expected (literal) target in the literal bias context versus the fixations to the expected (figurative) target in the figurative bias condition. The significant Bias × Sample interaction reflects the large difference between L1 and L2 groups for figurative targets given figurative bias contexts and more modest differences between L1 and L2 groups for literal targets given literal bias contexts (most apparent in Fig. 5B). The lack of interactions with *ot* terms and bias and sample indicate that similar polynomial forms were observed for both groups for both bias contexts (even though the forms differed by bias context). Simple effects GCA models confirmed that the effect of sample was significant for the figurative bias condition (*estimate* = −0.08, *CI* [−0.12, −0.03], *t* = −3.028, *p* =.002) but not for the literal bias condition (*estimate* = −0.01, *CI* [−0.06, 0.04], *t* = −0.404, *p* =.686).

**Anticipatory fixation proportions in the literal bias context.** For the anticipation window (prior to idiom onset), there is no need to assess the figurative bias context, as fixation levels to the expected (figurative) target in that window were similar to those of all the other items for both L1 and L2 participants. Thus, for this analysis, we focus on differences between the L1 and L2 samples in fixations to the expected (literal) target in the literal bias context. We began with a maximal model with four over-time (*ot*) parameters (i.e., orthogonal power-polynomials corresponding to slope, quadratic, cubic, and quartic terms). Equation 3 provides the model specification we provided to the R package *lme4* (Bates et al., 2015) to assess mean fixation proportions to the expected (literal) target in the literal bias context as a function of the four *ot* parameters as a set, the main effect of *sample* (L1, L2), and two-way interactions between each *ot* term and *sample*, along with maximal random effects structure.

3 $$\begin{array}{c}\text{meanFix }\sim (\mathrm{ot}1 +\text{ ot}2 +\text{ ot}3 +\text{ ot}4) \times \text{ Sample }+\\ (\mathrm{ot}1 +\text{ ot}2 +\text{ ot}3 +\text{ ot}4 |\text{ Subject})\end{array}$$

**Fig. 6:**
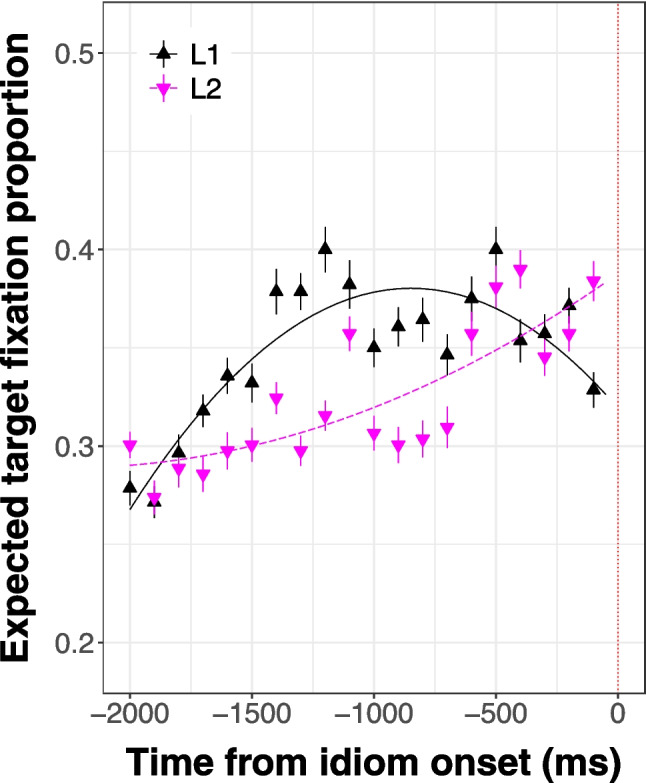
Anticipatory fixation proportions to the literal target in the literal bias context for samples L1 and L2. Points indicate mean fixation proportions. Lines indicate GCA fits. (Color figure online)

Neither the *ot3* nor *ot4* terms were significant, and did not significantly interact with *sample*. We then reduced the model in Equation 2 by removing the *ot4* terms. Again, the *ot3* term was not significant, and did not interact with *sample*. We then removed the *ot3* terms. The results from this final model are summarized in Table 5.^2^ Because *sample* was sum coded for contrasts, the *ot* main effects indicate how the mean polynomial form differed from a flat line with 0 intercept. The significant intercept indicates a robust difference from 0. The nonsignificant *ot1* term indicates mean slope did not vary significantly from 0. The significant *ot2* term indicates significant bowing. The interaction of *ot2* and *sample* indicates a difference between samples in the quadratic term. Figure 6 plots the mean fixation proportions and the GCA fits. From the figure, it is apparent that the quadratic aspect of the time series is quite different for the two groups. L2 shows a slight downward bowing (consistent with accelerating fixations towards the literal target) while L1 shows more salient upward bowing. As can be seen in Fig. 4, this reflects the tendency for L1 participants to look *away* from the literal target briefly at idiom onset.

**Table 5:** Anticipation (pre-idiom onset) GCA results for literal target fixations in the literal bias context for L1 and L2 samples

*Predictor*	*Estimate*	*CI*	*t*	*p*
(Intercept)	0.35	0.32, 0.38	**25.69**	**<.001**
ot1	0.11	−0.03, 0.25	0.13	0.122
ot2	−0.16	−0.27, −0.05	**−2.75**	**.006**
Sample [L2]	−0.02	−0.06, 0.01	−1.33	0.183
ot1 × Sample [L2]	0.06	−0.12, 0.25	0.67	0.501
ot2 × Sample [L2]	0.20	0.04, 0.35	**2.49**	**.013**

To confirm the source of the interaction, we assessed simple effects models for each sample. The results are summarized in Table 6, where we confirm a significant quadratic (*ot2*) effect for L1 but not for L2. We also see a significant effect of slope (*ot1*) for L2, but not for L1, consistent with the apparent trend for a positive slope for L2 in Fig. 6. However, because this was not sufficient to drive a significant *ot1 × Sample* interaction (see Table 5), no implications should be inferred.

**Table 6:** Summary of GCA tests of simple effects of *ot* terms for L1 and L2 for the anticipatory analysis

	**L1**				**L2**			
*Predictor*	*Estimate*	*CI*	*t*	*p*	*Estimate*	*CI*	*t*	*p*
(Intercept)	0.35	0.32, 0.38	**25.35**	**<,0.001**	0.32	0.30, 0.35	**26.47**	**<.001**
*ot1*	0.11	−0.04, 0.26	1.45	,0.148	0.17	0.06, 0.29	**2.86**	**.004**
*ot2*	−0.16	−0.28, −0.04	**−2.58**	**,0.010**	0.04	−0.06, 0.13	0.73	.467

#### Individual differences

We explored individual differences in the L2 group characterized by LexTALE scores for both the post-idiom onset window and the anticipatory window. Our sample size in the L2 group was rather small for individual differences analyses, so we emphasize that these are exploratory analyses. For presentation purposes, we include figures below based on a median split on LexTALE scores. For the lower half, the mean was 73.0, with a range of 62.5 to 82.5. For the upper half, the mean was 91.4, with a range from 83.8 to 98.8.

**Fig. 7:**
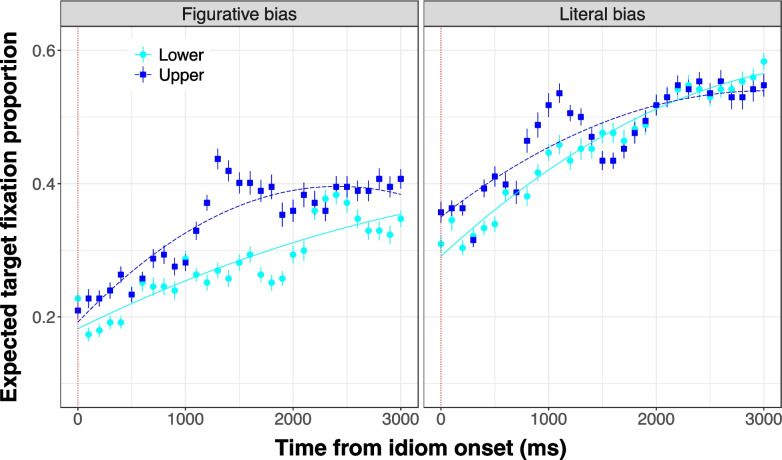
Post-idiom onset fixation proportions to expected targets in the figurative and literal bias contexts for L2 participants, divided by median split into Lower and Upper English ability halves based on LexTALE scores. Points indicate mean fixation proportions, lines indicate GCA fits. (Color figure online)

**Fig. 8:**
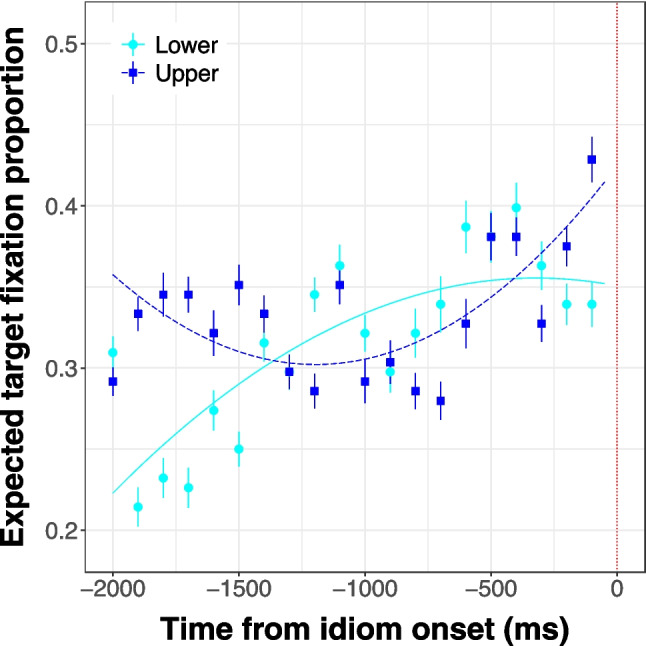
Anticipatory (pre-idiom onset) fixation proportions to literal targets in the literal bias context for L2 participants, divided by median split into Lower and Upper halves. Points indicate mean fixation proportions, lines indicate GCA fits. (Color figure online)

**Post-idiom onset*.*** For the post-idiom onset window, we first added LexTALE as a fixed-effect predictor to the GCA model. Results are summarized in Table 7, where we see a nonsignificant trend for LexTALE to have predictive power. Note that the statistical analyses were based on all L2 participants. In Figs. 7 and 8, to visualize trends across the L2 participants, we divided them into Lower and Upper English ability halves via a median split. The results are plotted in Fig. 7, where we see a (nonsignificant) trend for the Upper half to have higher fixation proportions in the figurative bias context.

**Table 7:** Individual differences in the post-idiom onset window for the L2 sample

*Predictor*	*Estimate*	*CI*	*t*	*p*
(Intercept)	0.04	−0.25, 0.33	0.26	0.792
ot1	0.21	−1.14, 1.56	0.31	0.759
ot2	0.42	−0.48, 1.31	−.91	0.364
Bias [Literal]	0.39	0.01, 0.78	**2.01**	**0.044**
LexTALE	0.00	−0.00, 0.01	1.84	0.065
ot1 × Bias [Literal]	0.79	−1.06, 2.64	0.83	0.404
ot2 × Bias [Literal]	−0.42	−1.58, 0.75	−0.70	0.482
ot1 × LexTALE	0.00	−0.01, 0.02	0.30	0.763
ot2 × LexTALE	−0.01	−0.02, 0.00	−1.17	0.244
Bias [Literal] × LexTALE	−0.00	−0.01, 0.00	−1.22	0.221
(ot1 × Bias [Literal]) × LexTALE	−0.01	−0.03, 0.01	−0.72	0.473
(ot2 × Bias [Literal]) × LexTALE	0.01	−0.01, 0.02	0.72	0.472

**Anticipatory window.** For the anticipatory (pre-idiom onset) window, we restricted the analyses to literal target fixations in the literal bias context as before, because there were no trends towards differences in the anticipatory window for the figurative bias context. We added LexTALE as a fixed-effect predictor to the GCA model. Results are summarized in Table 8, where we see a nonsignificant trend for LexTALE to have predictive power, as well as a nonsignificant trend towards an interaction of *ot2* and LexTALE. To visualize the results, we divided the L2 participants into Lower and Upper halves via median split. The results are plotted in Fig. 8, where we see a (nonsignificant) trend for the Upper half to have higher fixation proportions in the literal bias context. We also see a reversal of the quadratic trend for the Upper and Lower halves. However, these trends are both nonsignificant and theoretically opaque. Furthermore, if we joined the pre- and post-idiom onset windows (i.e., Fig. 8 and the right panel of Fig. 7), these quadratic differences would be damped considerably because the right panel of Figure 7 would appear as a continuous upward trend from Figure 8.

**Table 8:** Individual differences in the anticipatory window for the L2 sample (literal bias context only)

*Predictor*	*Estimate*	*CI*	*t*	*p*
(Intercept)	0.20	−0.01, 0.41	1.87	0.062
ot1	0.84	−0.20, 1.88	1.59	0.113
ot2	−0.72	−1.56, 0.11	−1.69	0.090
LexTALE	0.00	−0.00, 0.00	1.17	0.242
ot1 × LexTALE	−0.01	−0.02, 0.00	−1.27	0.205
ot2 × LexTALE	0.01	−0.00, 0.02	1.79	0.074

To summarize the individual differences analyses, we observed nonsignificant trends of varying theoretical interest. In the anticipatory window, participants with higher LexTALE scores showed a nonsignificant trend towards higher fixations proportions to the literal target. In the post-idiom onset window, participants with higher LeXTALE scores tended to fixate the figurative target more than did participants with lower LexTALE scores, although again this trend was nonsignificant. Of note here is the salient resemblance between Figure 8, showing anticipatory fixations to literal targets in the literal bias condition for L2 participants, divided by median split into Lower and Upper halves, and Fig. 6, showing anticipatory fixations to the literal target in the literal bias condition for L1 and L2 participants. Visual comparison of these two figures reveals that the “upper” median split group in Fig. 8 shows the same trend as L1 participants in Fig. 6, whereas the “lower” median split group shows the same trend as L2 participants. We will return to these results in the Discussion.

## Discussion

The present study investigated comprehension of figurative formulaic expressions in L1 and L2 English speakers. First, we asked whether figurative expressions are activated and retrieved qualitatively differently than literal expressions with similar collocational frequency. Second, we asked whether L1 and L2 speakers relied on different strategies (compositional processing vs. holistic retrieval) when accessing idiom forms. To address these questions, we conducted growth-curve analyses of gazes to the predicted targets in the literal and figurative bias conditions in two time-windows, one of anticipation driven by the context sentence, and a later window in which eye-movements were driven by the target expression. Our results make two clear contributions to our understanding of figurative language comprehension in native and nonnative speakers. First, we observed a significant Bias × Sample interaction reflecting a magnification of L1 and L2 differences for idiom processing. Second, we observed a strong anticipatory effect for literal targets in the literal bias condition that was not present for figurative targets and which varied based on population. We discuss these results now in detail.

We turn first to our second research question, which asked whether L1 and L2 speakers relied on different processing strategies during idiom comprehension. Examination of the post-idiom onset time window provides suggestive evidence that L1 and L2 speakers are indeed biased towards different processing strategies. L2 speakers differed significantly from L1 speakers when anticipating figurative targets: although the curve of fixation proportions towards the figurative target did not significantly differ between populations, L2 speakers had significantly lower figurative target fixation proportions in figurative, but not literal, bias conditions. The tendency for L2 speakers to have lower target fixation proportions than L1 speakers (Fig. 5) and lower figurative target selections (Fig. 2) in the figurative condition may indicate less automatic processing of idioms, or even a tendency towards initially processing idioms in a “literal mode.” This is congruent with previous accounts of a bias towards compositional analysis during L2 idiom comprehension and processing (Carrol et al., 2016; Senaldi & Titone, 2022). Note that this pattern does not reflect lower proficiency on the part of our L2 speakers in general: our L2 speakers had high enough proficiency to match L1 speakers in how quickly and efficiently they processed literal condition items (Fig. 5). They even showed similar literal target anticipation as L1 speakers in the literal condition. Rather, this pattern is specific to anticipating and accessing figurative forms.

In contrast, we predicted that L1 speakers would be biased towards an automatic holistic retrieval processing strategy, leading to a higher proportion of fixations to the figurative target in inappropriate contexts (Titone et al., 2019). We did not see evidence of such a strategy. Instead, L1 speakers showed highly successful contextually appropriate processing, with no apparent effects of interference from contextually inappropriate targets or the two lures.

It is worth pointing out here that the nature of the present experiment may have biased participants away from direct retrieval and towards a more compositional processing strategy. This is because targets related to the literal meanings of the idioms’ final words were directly presented to participants as possible completions in all conditions (Kyriacou & Köder, 2024). Use of the visual world paradigm has significant advantages: It enables close evaluation of the cognitive processes underlying idiom comprehension and yields detailed information about the time-course of these processes, and has been used successfully in previous studies investigating idiom comprehension (Holsinger, 2013; Kessler et al., 2021). However, visually presenting targets related to the literal meanings of components in the idioms may have pushed participants towards a more compositional processing strategy. This bias was evident in Holsinger’s (2013) visual world study of idiom comprehension, in which participants looked at visually presented semantic associates of idioms more than unrelated control words. For example, when listening to *kick the bucket,* participants looked at FOOT more often than TRIANGLE. This raises the possibility that simultaneous visual presentation of literal alternatives can trigger idiom decomposition processes which in turn lead to activation of semantic associates through spreading activation. It is an open question to what degree these processes are in play during naturalistic idiom comprehension (Kyriacou & Köder, 2024). If this is the case, it is not surprising that any predicted effects of context-inappropriate reliance on direct retrieval by L1 participants in the present study would be eclipsed by a stronger experiment-specific bias towards compositional processing. The balance of the evidence is still that, generally, L1 speakers are biased towards a direct retrieval strategy when comprehending idioms (Carrol & Conklin, 2020; Conklin & Schmitt, 2008; Libben & Titone, 2008; Tabossi et al., 2009). This bias and its consequent effects on eye movements to targets in inappropriate contexts may not have been entirely apparent given the design of the present study.

Consider our first research question, which asked whether figurative expressions are activated and retrieved qualitatively differently than literal expressions with similar collocational frequency. Eye movement patterns, quantified by the growth curve results (Fig. 4), were dramatically different between literal and figurative bias conditions, suggesting qualitative activation and retrieval differences between literal and figurative expressions. In particular, literal expressions showed robust anticipatory processing, with the target object being strongly fixated during the anticipatory window by both groups. However, we also saw evidence of group differences in this anticipatory window (Fig. 6), as indicated by a significant Quadratic Component × Sample interaction: L2 speakers showed a patterns of accelerating fixations towards the literal target, whereas L1 speakers looked away from the literal target briefly at phrase onset. The brief decline in fixations for L1 speakers near idiom onset may suggest a return of attention to the bottom-up signal, whereas the steady increase in fixations for L2 speakers may indicate stronger reliance on context to drive eye movements for this population. In contrast, fixations to the target figurative object in the figurative condition did not increase until the later, nonanticipatory, window, when participants were able to use the target expression to direct gaze.

It is worth considering possible drivers of prediction in the literal bias condition, and why any such drivers of prediction were not at play in the figurative bias condition. One possibility is that, in the literal bias condition, participants had access to event-based knowledge (knowledge about how the world works (McRae & Matsuki, 2009). When producing language, we tend to talk about things that are likely to happen, and therefore our knowledge about both word co-occurrences and how the world works converges, allowing us to predict upcoming words (Altmann & Kamide, 1999; Borovsky et al., 2012; Kamide et al., 2003; Kukona et al., 2011, 2016). For example, recall the example image array shown in Fig. 1, showing the figurative target (table), literal target, (car), high-collocation lure (key), and low-collocation lure (flask). In the literal bias condition, participants may have found it easy to access knowledge about *traveling* events upon hearing the contextual biasing phrase “To get to his destination,” and therefore predicted that the appropriate target would be the *car* before even hearing the target phrase. This information may not have been available in the figurative bias condition: it seems difficult to predict the presence of “tables” given “To get even with his enemies.” Taken together, although the final word of an idiom may be extremely predictable given the first few words of that idiom (see Sprenger et al., 2024, for evidence that early lexical units of an idiom can prime later lexical units), the upcoming *presence* of an idiom given a particular context is not.

However, this explanation does not rule out an assistive effect of figurative contexts. Rather than allowing the comprehender to predict the final word of the idiom, context may instead help the comprehender narrow down their choices in the later time window. The present experiment does not allow us to distinguish between these possibilities. However, it is clear that idiom identification proceeds rapidly once the first few words of the idiom have been presented. Furthermore, this behavior dovetails well with a recent perspective proposed by Colston and Gibbs (2021): Far from being a disadvantage, the inherent indirectness of figurative language is instead an efficient vehicle for critical pragmatic functions of communication that “normal” compositional language is not able to achieve. Language users make specific choices to use figurative language because this type of language is most effective at capturing the complexity of thought, packaging it into a concise linguistic bundle, and conveying meaning to others.

Finally, we turn to a brief discussion of the exploratory individual difference analyses. This evidence suggests that, despite the high proficiency of our sample, L2 speakers’ figurative competence was nonetheless still developing as proficiency increased. The exploratory individual differences analyses showed a nonsignificant trend for higher-proficiency speakers to have higher target fixation proportions in the figurative bias context (Fig. 7). This mirrored the fixation patterns of L1 speakers, suggesting that figurative competence was developing as proficiency increased. It should be stressed that our sample size was quite small for individual differences analyses, so these results are emphatically exploratory. Additionally, our sample of L2 speakers was highly proficient. Nevertheless, it is notable that we were able to observe distinct patterns of figurative competence developing in such a proficient sample of L2 speakers. These exploratory results suggest future avenues of research investigating the trajectory of idiom acquisition in more diverse samples of adult L2 speakers.

In a previous publication using the same data from L2 speakers (Milburn et al., 2021), we showed that higher English proficiency (indexed by higher LexTALE scores) aided L2 speakers by allowing them to more easily ignore inappropriate figurative targets in the literal bias condition. In that instance, higher proficiency helped speakers not by facilitating processing of figurative material in supportive contexts, but rather by increasing speakers’ ability to suppress inappropriate figurative lures in inappropriate contexts. It is worth asking, then, if this pattern is evidence of L2 speakers developing more native-like processing strategies as proficiency increases, or if this pattern reflects a successful, but uniquely L2 processing strategy. In the current paper, L1 speakers showed no evidence of increased suppression of figurative targets in inappropriate contextual conditions. Rather, their gaze patterns showed precise contextually appropriate processing. Increased L2 proficiency also appeared to confer increased native-like processing, with higher-proficiency L2 speakers as indexed by a nonsignificant trend towards higher proportions of fixations to the figurative target in the figurative bias condition in the post-idiom bin (Fig. 7) by higher-proficiency L2 speakers (note that “higher-proficiency” here is relative, as all L2 speakers in the present study were high proficiency), paralleling the fixation patterns of L1 speakers.

## Conclusions

The present study found evidence that L2 speakers may be biased towards compositional processing of idioms (in contrast to more holistic idiom processing by L1 speakers), with indications that figurative competence is still developing even among highly proficient L2 speakers. We also found evidence of qualitative processing differences between literal and figurative expressions, with literal processing being strongly driven by anticipatory mechanisms and figurative processing being driven by within-phrase associations. These results illustrate the complex interactions between contextual bias and internal idiom predictability, and pave the way for future investigation of the developmental trajectory of idiom acquisition.

## Data Availability

All (deidentified) data and materials are available (https://osf.io/7cyus/overview). This study was not preregistered.

## Appendix

Table 9

**Table 9:** Linguistic stimuli, target and distractor images, and COCA frequencies

Condition	Context	Idiom	Fig. Target	Lit. Target	High-Coll. Lure	Low-Coll. Lure	L2 Fam.	L1 Fam.
			/COCA Freq.	/COCA Freq.	/COCA Freq.	/COCA Freq.	(Min-Max [Mean])	(Min-Max [Mean])
Figurative	James's big deadline was looming, so yesterday he	broke his	back	foot	neck	clarinet	1-7 [3.4]	1-6 [2.05]
Literal	The doctor gave James crutches because yesterday he		0.05	0.04	0.15	0		
Unbiased	Yesterday he							
Figurative	Anna was such a great boss that no one could	fill her	shoes	glass	eyes	suitcase	1-4 [1.7]	1-7 [1.85]
Literal	At dinner Anna was angry because no one could		0.01	0.01	0.04	0		
Unbiased	No one could							
Figurative	At the wild party last night, Carol	raised the	roof	knife	gun	iron	1-7 [2.4]	1-6 [1.95]
Literal	When making dinner, Carol		0.02	0.02	0.08	0		
Unbiased	Carol							
Figurative	To convince Cathy, Amy	twisted her	arm	mouth	head	apron	1-7 [2.5]	1-6 [1.55]
Literal	The lemon was sour, so Amy		0.02	0.03	0.04	0		
Unbiased	Amy							
Figurative	In her relationship, Silje wanted to	wear the	pants	mask	crown	flip flops	1-7 [2.8]	1-5 [1.45
Literal	For her disguise, Silje wanted to		0.04	0.04	0.05	0		
Unbiased	Silje wanted to							
Figurative	After initial fame, the actor eventually	fell off the	map	table	wall	treadmill	1-7 [2.35]	1-6 [1.8]
Literal	Unfortunately, the valuable teapot eventually		0.01	0.01	0.03	0		
Unbiased	Khalil eventually							
Figurative	Because she was exhausted, Victoria	hit the	sack	dirt	road	iceberg	1-5 [1.55]	1-2 [1.15]
Literal	After tripping on a rock, Victoria		0.09	0.09	1.25	0.03		
Unbiased	Victoria							
Figurative	If you want to negotiate successfully, you have to	hold the	cards	pen	door	fork	1-6 [3.15]	1-7 [2.85]
Literal	If you want to write a letter, you have to		0.02	0.02	0.19	0.01		
Unbiased	You have to							
Figurative	Raj was eager to get started, but he had to	hold his	horses	baby	hand	pencil	1-7 [2.15]	1-3 [1.45]
Literal	In the hospital, Raj had to		0.01	0.01	0.17	0		
Unbiased	Raj had to							
Figurative	Omar's grandmother was about to	kick the	bucket	door	ball	garbage	1-7 [2.15]	1-4 [1.2]
Literal	To get into the room, Omar was about to		0.05	0.06	0.11	0		
Unbiased	Omar was about to							
Figurative	Samir loved to tease Anja, and always	pulled her	leg	bike	hand	tooth	1-7 [2.3]	1-4 [1.3]
Literal	To bring it inside, Anja always		0.01	0.01	0.2	0		
Unbiased	Anja always							
Figurative	After a long and exhausting day, Bianca had	run out of	juice	motor oil	money	socks	1-6 [2]	1-3 [1.5]
Literal	In the garage, Bianca had		0.04	0.05	0.56	0		
Unbiased	Bianca had							
Figurative	No one wanted to be responsible, so Lizzy had to	take the	wheel	house	bus	hat	1-3 [1.2]	1-3 [1.2]
Literal	The real estate agent said that Lizzy had to		0.13	0.13	0.42	0.01		
Unbiased	Lizzy had to							
Figurative	At the baseball game, Sai had to	warm the	bench	engine	cooking oil	camera	1-7 [3]	1-7 [2.05]
Literal	Before going to work, Sai had to		0.01	0.01	0.03	0		
Unbiased	Sai had to							
Figurative	After one too many mistakes, Radhi finally	got the	ax	tattoo	phone	paint	1-7 [4.2]	1-7 [2.4]
Literal	On her 18th birthday, Radhi finally		0.02	0.02	0.16	0.01		
Unbiased	Radhi finally							
Figurative	Pamela often talked back to her boss and loved	playing with	dynamite	computers	dolls	pigeons	1-7 [3]	1-7 [3.3]
Literal	The students in the technology class loved		0.01	0.01	0.08	0		
Unbiased	Pamela loved							
Figurative	When he was almost out of money, Pablo	tightened his	belt	hands	lips	shoe	1-7 [4.1]	1-7 [2.1]
Literal	Before throwing the punch, Pablo		0.01	0.01	0.03	0		
Unbiased	Pablo							
Figurative	To get even with his enemies, Chia-Ming	turned the	table	car	key	flask	1-3 [1.35]	1-6 [2]
Literal	To reach his destination, Chia-Ming		0.19	0.17	0.4	0.01		
Unbiased	Chia-Ming							
Figurative	To do well on the test, Li Yun	hit the	books	car	beach	couch	1-7 [1.8]	1-3 [1.3]
Literal	Although she tried to swerve, Li Yun		0.09	0.07	0.17	0.01		
Unbiased	Li-Yun							
Figurative	In the puzzle game, Asha	held the	key	baby	door	spatula	1-7 [2.8]	1-6 [2.4]
Literal	While the family was busy, Asha		0.1	0.11	0.46	0		
Unbiased	Asha							

Figurative and literal targets were matched in collocation frequency using the Corpus of Contemporary American English (COCA; Davies, 2009). Using COCA’s Collocates function, we searched for noun collocates of the idiom with the final word missing (i.e., noun collocates of “turned the”), spanning two words to the right. For literal targets, we selected noun collocates that were concrete, imageable, and were similarly frequent to the figurative target. We also selected high and low collocation frequency lure items.

Stimuli were normed for familiarity by both L1 and L2 speakers. Participants rated how familiar they were with idioms using a 1-7 Likert scale (1 = very familiar; 7 = very unfamiliar). Note that under this measurement, lower ratings indicate greater familiarity with an idiom.

## Footnotes

1 While the patterns of significance did not change when *ot4* and/or *ot3* terms were included, those models did account for significantly more variance than the final model. We chose the model with only the *ot1* and *ot2* time terms to make clear the impact of those terms in the model-data comparison figure below (Fig. 5). Including the *ot3* and *ot4* terms makes the GCA fits conform slightly better to the points, but the differences are not interpretable, nor are they of theoretical interest.

2 As with the previous analysis, the patterns of significance did not change when *ot4* and/or *ot3* terms were included, although those models did account for significantly more variance than the final model. We chose the model with only the *ot1* and *ot2* time terms to make clear the impact of those terms in the model-data comparison in Fig. 6. Including the *ot3* and *ot4* terms makes the GCA fits conform slightly better to the points, but the differences compared to the analysis we present here are not interpretable, nor do they have theoretical interest.

## Code availability

Code used for data analysis is available (https://osf.io/7cyus/overview).
